# Experimental and computational evaluation of cyclic solvent injection in fractured tight hydrocarbon reservoirs

**DOI:** 10.1038/s41598-021-88247-y

**Published:** 2021-05-04

**Authors:** Amin Ghanizadeh, Chengyao Song, Hamidreza Hamdi, Christopher R. Clarkson

**Affiliations:** Department of Geoscience, University of Calgary, Calgary, Canada

**Keywords:** Core processes, Environmental impact

## Abstract

Multi-fractured horizontal wells have enabled commercial production from low-permeability (‘tight’) hydrocarbon reservoirs but recoveries remain exceedingly small (< 5–10%). As a result, operators have investigated the use of solvent (gas) injection schemes, such as huff-n-puff (HNP), to improve oil recovery. Previous HNP laboratory approaches, classified primary as ‘flow-through-matrix’ and ‘flow-around-matrix’ typically (1) are not fully representative of field conditions at near-fracture regions and (2) require long test times, even when performed on fractured cores. The objectives of this proof-of-concept study are to (1) design and implement a new experimental procedure that better reproduces HNP schemes in near-fracture regions and (2) use the results, simulated with a compositional lab-calibrated model, to explore the controls on enhanced hydrocarbon recovery in depleted tight oil plays. Performing multiple CO_2_ and (simplified) lean gas HNP cycles, the integrated experimental and simulation approach proposed herein achieves the ultimate recovery factors in a significantly shorter time frame (25–50%) compared to previous studies. The integrated experimental and computational approach proposed herein is valuable for core-based evaluation of cyclic solvent (CO_2_, CH_4_) injection in tight hydrocarbon reservoirs for (1) hydrocarbon recovery and (2) subsurface greenhouse (CO_2_, CH_4_) gas disposal/storage applications.

## Introduction

Hydrocarbon liquid-rich shale plays are currently the hottest unconventional reservoir targets in North America. However, the majority of development has focused on primary depletion with multi-fractured horizontal wells, and hydrocarbon liquid recovery is projected to be low (˂5–10%). Further, the current recovery process is inefficient in the majority of tight oil plays, and does not offer a solution to reducing greenhouse gas emissions. A potential solution is to inject CO_2_, or reinject produced (lean/rich) gas recovered from the reservoir, or a combination of both, back into multi-fractured horizontal wells for the purpose of simultaneously increasing liquid hydrocarbon recovery (condensate or oil), and reducing greenhouse gas (CO_2_, CH_4_) emissions. The cyclic solvent injection process, commonly referred to as huff-n-puff (HNP), with CO_2_ and/or lean gas as the ‘solvent’, is a potentially attractive mechanism for co-optimization of greenhouse gas sequestration and enhanced oil recovery (EOR) because, unlike solvent (e.g. CO_2_) flooding and water-alternating-gas flooding (e.g. CO_2_-WAG) (dedicated injection and producing wells) scenarios, large expenditures on specialized facilities, in-field pipelines and well conversions are unnecessary. Despite these advantages, industry requires critical data, evaluation methods, and supporting simulation studies in order to make a decision on whether to invest capital in piloting the HNP process in their operated wells/fields.

A summary of the theory behind the HNP process in tight hydrocarbon systems is provided in Supplementary Appendix S1 online. There are now numerous studies of improved oil recovery in a variety of North American tight oil reservoirs that have examined the feasibility of incremental oil recovery using gas injection^1–6^. These studies have primarily focused on numerical^2,3,6^ or laboratory-based approaches^1,7–9^. Laboratory-based studies are important for assessing (1) the key mechanisms controlling injected gas transport into the reservoir, and miscibility with the oil^7,8,10^ and (2) the influence of operational parameters including injection pressure/time, soaking time, production pressure/time—among other factors (e.g. PVT)—on recovery performance^7,11^. Further, laboratory-based analyses are required to (1) understand the underlying physics and (2) constrain simulation models used for HNP schemes. By matching the laboratory results (e.g. recovery factors, oil/gas production) using rigorous fine-scale numerical simulation models, critical parameters that can be used in evaluating field-scale EOR pilots may be obtained.

The experimental studies of cyclic solvent injection in tight hydrocarbon systems performed to date are extensive, providing much insight into the fundamental mechanisms and controlling factors, but have some limitations (Supplementary Table S1 online). The majority of previous laboratory studies have been performed on core plug samples with permeability values down to the microdarcy/millidarcy range^12,13^ until now, very few laboratory-based studies^8,14–17^ have been conducted on tight hydrocarbon systems with permeability values down to the nanodarcy range (a common permeability range for unconventional hydrocarbon reservoirs). Further, the laboratory methodologies are primarily focused on intact (‘unfractured’) core plug samples using either the ‘flow-through-matrix’^12,18,19^ or ‘flow-around-matrix’^1,20,21^ schemes, which do not realistically represent the fracture-matrix contact or sequences associated with a typical cyclic solvent injection (HNP) process. While seminal works have been conducted to mimic HNP process on fractured cores^7,8^, the fractures were created outside of the coreholder either fully using saw^7^ or partially using drilling bits^8^ (combined with hydraulic fracturing under stress inside coreholder). These approaches, though more representative, are prone to the creation of stress-induced micro-fractures, particularly in organic-rich shales (the primary target plays for field-scale HNP application). If present, these micro-fractures, the apertures of which may be below the detection limit of CT/micro-CT scan particularly for larger cores, could act as ‘by-pass’ flow paths along and in between the primary fractures, causing overlap between different mechanisms. The representative replication of *HNP* mechanism, de-coupled from *flooding* mechanism, is a challenge in experimental HNP studies. Multi-fracture geometries, though more representative of subsurface condition, may provide a combination of flooding and HNP mechanisms under laboratory conditions with an elevated effective permeability due to the proximity between saw-cut fractures or hydraulically-induced primary fracture and the artificially-induced micro-fractures. In addition, previous laboratory-based HNP experiments were typically not simulated using a compositional (lab-calibrated) model. More importantly, the experimental durations of previous laboratory techniques are extensively long, even for fractured cores (typically 15–20 h per cycle^7,8^). This limitation hinders the experimental throughput, and the capacity for characterizing the HNP mechanisms/controls over diverse experimental conditions in a timely fashion.

**Figure 1:**
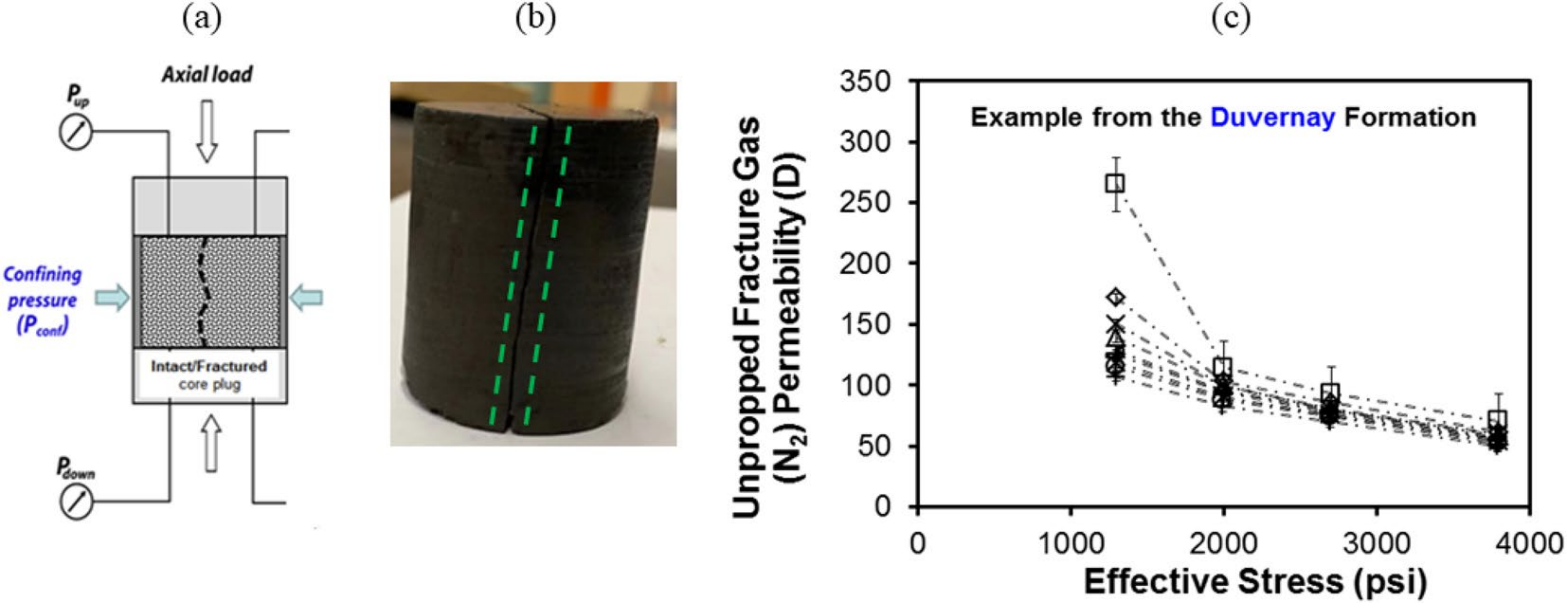
Use of biaxial coreholder to induce fractures in core plugs under stress and measure gas fracture permeability **(a)**; example of core plug sample (Duvernay Formation, western Canada) fractured under differential stress in biaxial coreholder **(b)**; example of unpropped fracture permeability measurements **(c)**. Modified after Ghanizadeh et al.^23^ and Zhang et al.^24^.

In order to represent the core-based HNP process at ‘near-fracture’ conditions from *locally* *depleted* tight oil reservoirs, a new set of experiments are designed herein whereby core-flooding is performed on core plug samples that have been artificially fractured in a process analogous to “in-situ” fracturing. In previous work by Ghanizadeh et al.^22,23^, it was demonstrated that (gas) fracture permeability could be measured on the intact core plug samples after artificial fractures were created in the rock under stress through application of differential stress in a conventional biaxial coreholder (Fig. 1). This method is designed to create the fracture *inside* the coreholder under stress by gradually increasing the axial load while keeping the radial load constant, until an axial splitting (i.e. fracture) is induced in the core plug (presumably) along the weakest plane (e.g., existing bedding lamination, etc.). While this approach (biaxial rather than shear) does not fully capture the complexity of natural fractures induced under sub-surface conditions, it is expected to mimic the “in-situ” fracturing conditions (i.e. fracturing under stress) better compared to the conventional methods (e.g., Brazilian test) in which the fracture is created artificially *outside* of the coreholder using uniaxial stress. This approach has enabled the measurement of tight rock unpropped/propped fracture permeability/conductivity^23^, and fracture compressibility^24^ under “in-situ” stress and as a function of effective stress.

In this work, a new workflow is proposed for core-based HNP experiments in fractured core plug samples as follows (Supplementary Fig. S1 online): (1) an intact core plug sample is first fractured under stress through application of differential stress inside the coreholder (no use of saw or drill bit); (2) the fractured core plug is saturated with oil by re-injecting formation oil back into the sample using liquid pump; (3) gas (e.g. CO_2_, lean gas) is injected into the sample and allowed to soak, to mimic the injection and soaking periods of a field-scale HNP scheme; (4) the injected gas is then re-injected in the upstream end at high pressure, and the effluent is measured at the downstream end. This set of experiments is expected to be more representative of ‘near-fracture’ conditions because (1) gas and liquid flow occurs through an actual “induced” fracture along the core plug sample, and (2) “in-situ” stress, and stress variation regimes during injection, soaking and flow is reproduced.

Core-flooding experiments require careful design in terms of the length of the injection, soaking and production periods, injected fluid volumes and pressures, and frequency of effluent sampling, among other considerations. Core-based simulation, when combined with actual measurements, has proven to be significantly helpful in our previous studies for developing new fluid flow experimental techniques^25,26^ and evaluating the impact of micro-scale heterogeneities on lab-based petrophysical and geomechanical data^27^. One of the novelties of this work is the optimization of the experimental HNP design by simulating the experiments using a compositional numerical simulator, with the core plug as the reservoir, prior to the actual tests. The simulator takes into account the various mechanisms (e.g. multi-phase advection through fracture, diffusion through matrix, oil solvation, etc.) that are expected in a core-based HNP experiment. Simulation of the experiments beforehand “de-risks” the tests, improving the chance of success (i.e. higher recovery). This aspect is beneficial for experimental design, particularly considering the overwhelming number of possible operational combinations (e.g. injection/production pressure/time, soaking time, gas type, etc.) that can be chosen for a given core-based HNP test. Apart from experimental design optimization, compositional numerical simulation is used in this work to history-match each cycle, enabling the fundamental controlling mechanisms to be explored after the experiments are completed. As a continuation of a previous work focused on CO_2_ HNP^28^, the current study is the first to couple experimental and modeling approaches for core-based comparison of CO_2_ and (simplified) lean gas HNP performance in low-permeability fractured reservoirs.

## Numerical simulation: prior to experiments

In order to assist with the experimental design prior to conducting the actual tests, and to select the optimum experimental conditions (leading to highest recovery), numerical simulations were implemented to simulate the expected oil recovery during the first four cycles of CO_2_ HNP experiments. Based on previous laboratory fluid analysis results^6^, a 7-component equation of state (EOS) was developed using tNavigator (version 20.1: https://rfdyn.com/tnavigator/). The mole fractions of the fluid components in the model were obtained by performing a two-phase flash to represent the dead oil sample used in the experiment. The composition of flashed oil at room conditions (1 atm, 25 °C) indicates that the majority of components are C5 to C12 (Supplementary Table S2 online). The phase envelope of the (simulated) dead oil is shown in Supplementary Fig. S2 online.

A numerical model was built prior to the CO_2_ HNP experiment using the petrophysical properties of the intact^29^ and fractured^24^ twin core plug samples to represent the matrix and fracture system in the simulation (Supplementary Fig. S3 online). This cuboid model was designed to have a similar surface area and pore volume as the actual cylindrical core plug samples used in this study. The model included 20 × 1 × 18 global cells in the x, y and z directions, respectively. The fracture-hosting cells were refined by logarithmic spacing to accurately model the flow towards the surface of an embedded fracture with a width of ~ 0.003 cm. The simulations were conducted using an effective gas diffusion coefficient (*D*) of 7 × 10^–4^ cm^2^/s for the CO_2_ component only^30^. The oil effective diffusion coefficient was set to be an order of magnitude smaller than the gas diffusion coefficient. Additionally, a uniform initial oil saturation = 100% and matrix porosity = 2.8% were assumed for the core plug sample. The stress-dependent matrix^29^ and fracture (formation oil) permeability (Supplementary Appendix S2 online), both derived from laboratory analysis, were employed to further constrain the flow simulations.

Nearly 1000 compositional simulation runs were performed to determine an optimal design for the CO_2_ HNP experiment. The parameters were selected using manual sensitivity analysis with the objective of optimizing the performance of core-based huff-n-puff process (i.e. higher oil production/recovery over shorter period). The effective diffusion/dispersion coefficients and the fracture aperture were selected as the primary input parameters for optimizing the experimental conditions because they were highly uncertain and the most impactful on oil production/recovery. Matrix permeability was also adjusted slightly but only within the range previously reported for other (Duvernay shale) twin core plug samples with similar porosity and organic/inorganic composition^31^. Two-phase (gas/liquid) relative permeability data, obtained for tight siliceous samples^29^ from the Western Canadian Sedimentary Basin were also among the initial selected parameters to constrain the simulation. However, for near-miscible scenarios (the case herein), its impact was determined to be insignificant based on repeated simulations (CO_2_) using another set of relative permeability curves obtained for other tight formations^32^. The numerical simulations were used to determine Minimum Miscibility Pressure (MMP) between CO_2_ and the “in-situ*”* (dead) oil, as well as the duration of gas injection, injection pressure, soaking and production periods. As for the simplified lean gas HNP tests, similar experimental conditions (i.e. injection time/pressure; soaking time; and production time) were applied without performing the “prior to experiment” simulations, in order to obtain a direct comparison between the two HNP EOR schemes (CO_2_ vs. simplified lean gas). In addition to experimental design, the simulation models used for experimental design were also used to history match the experimental oil recovery data after the actual experiments with CO_2_ and simplified lean gas were completed.

While ideal, there are considerable numerical complexities/challenges associated with using small grid blocks in the available commercial reservoir simulators for *miscible/near-miscible* scenarios where diffusion/dispersion is included. Using a fully implicit scheme to include the effective diffusion/dispersion coefficient as an input, precautions were taken when selecting the grid blocks to (1) ensure the gridding is small enough to effectively capture the associated physics and avoid numerical dispersion as much as possible, and, (2) avoid simulation failure due to very small grid blocks. The small explicit fracture in the numerical simulation model was represented by grid blocks as small as 0.003 cm that can be optimally simulated (i.e. without any failure) by the available commercial reservoir simulators. The near-fracture grids were further refined to around 0.04 cm with larger grids up to a maximum of ~ 0.18 cm in the regions far away from the fracture.

## Experiments

### Experimental setup

**Figure 2:**
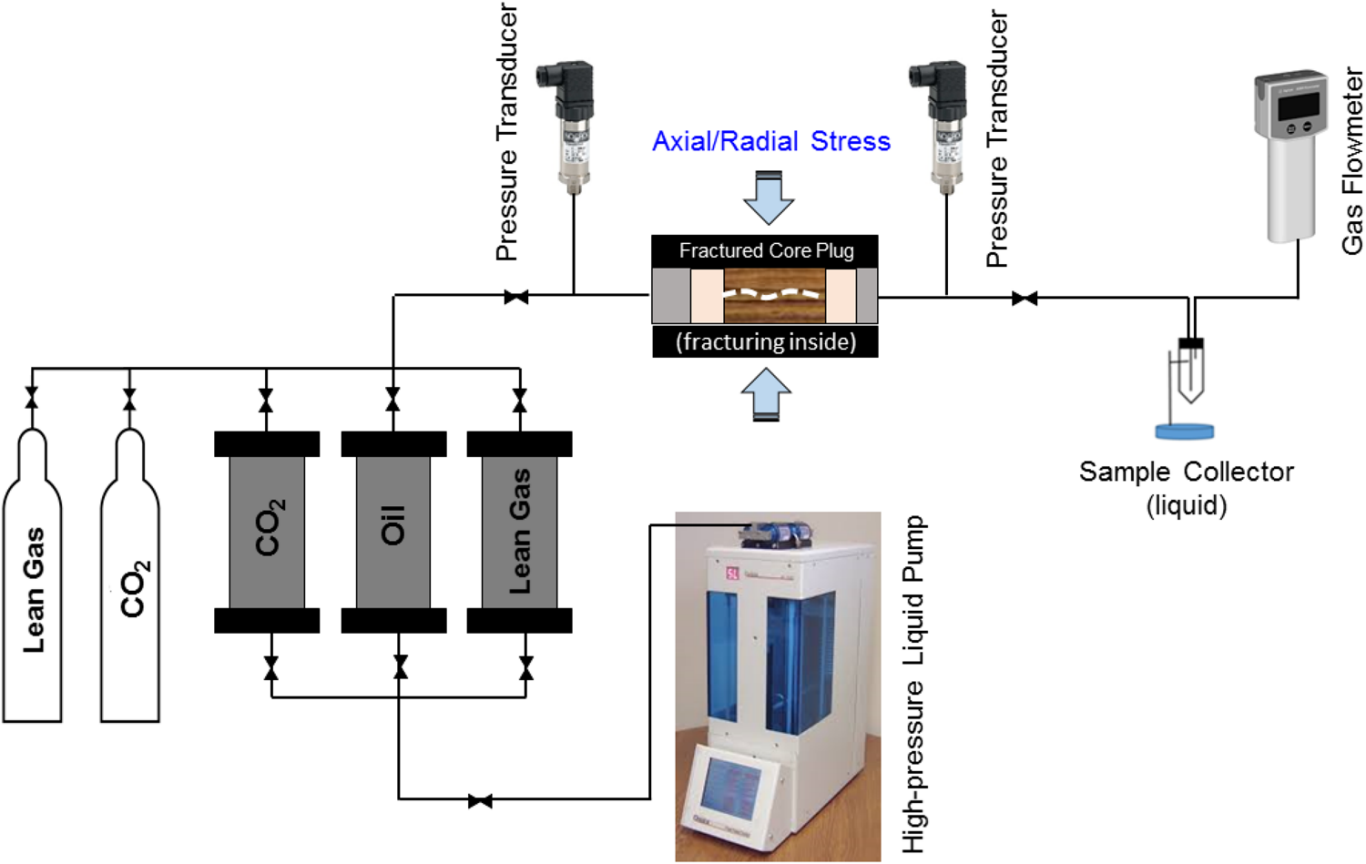
Schematic of the experimental setup designed and built in-house for the cyclic solvent (gas) injection (HNP) experiments.

The experimental setup for the cyclic solvent (gas) injection includes a high-pressure fluid injection pump, gas (CO_2_, lean/rich gas) cylinder, fluid transfer cylinders, high-pressure coreholder, pressure transducers, produced oil sample collector, gas flowmeter, gas sampling cylinders and various valves to control the flow in and out of the coreholder (Fig. 2). The coreholder is capable of applying a confining pressure up to 10,000 psi to mimic in-situ stress conditions. The confining pressure can be applied in radial and axial directions, independently (i.e. biaxial coreholder). Using the high-pressure (5000 psi) pump, oil and liquefied CO_2_ stored in the transfer cylinders can be injected into the coreholder at either constant flow rate or constant pressure conditions to perform liquid permeability and HNP experiments. The injection pressure and production pressure between two ends of the coreholder are monitored and recorded by two separate pressure transducers with an accuracy of ± 0.025% of full scale (0–6000 psi). The produced liquid and gas samples are separated in the oil sample collector that consists of a measurement tube with graduated volume (0.01 cc). Cumulative oil production is recorded using the sample collector while the gas production is measured using a gas flowmeter (0–1,000 ml/min flow rate range). The produced gas samples are collected in a gas sampling cylinder (0–500 psi pressure range) during HNP experiments for compositional analysis. In this experimental setup, the downstream dead volume was comprised of the volume within a capillary tube (0.55–0.8 cc) and the dead volume (~ 0.20 cc) associated with a needle valve (technically inevitable).

### Experimental workflow and procedure

The key steps/experiments for core-based HNP, reproduced from Song et al.^28^, were as follows:

1. The liquid permeability device used for steady-state liquid flow tests was calibrated using previously-designed in-house permeability standards (micro-capillaries embedded into impermeable acrylic plugs, references within Song et al.^28^). To pass the calibration, the measured permeability values should be within ± 15% of the calculated permeability value.
2. A core plug sample with a cemented lamination occurring its entire length was selected (see “Rock sample preparation: fracturing under stress”).
3. Helium porosity was measured on the core plug sample (using the helium pycnometry technique combined with calipered dimensions).
4. The core plug sample is assembled into the core-holder and estimated “in-situ” stress is applied, followed by a leak test at about 500 psi higher that the highest working pressure for HNP tests.
5. A fracture was created along the length of the cemented lamination artificially inside of the coreholder by keeping the radial stress constant while increasing the axial stress stepwise—this approach is generally expected to result fracture creation under stress which is more similar to the subsurface conditions, compared to creating fractures outside the coreholder, as discussed previously^22,23^.
6. Unpropped fracture permeability and compressibility with gas (N_2_, CH_4_) was measured under loading (i.e. stepwise increase of effective stress; representative of primary depletion) and unloading (i.e. stepwise decrease of effective stress; representative of injection) paths using the steady-state gas flow technique. The gas permeability values were measured after each incremental increase in axial/radial stress conditions and repeated within 12-h time intervals under similar experimental conditions until a constant value (within experimental error range) was achieved—the axial/radial stress should be increased (during loading) or decreased (during unloading) only after constant permeability values are reached (as a result of ‘stress creep’). Details of unpropped/propped fracture permeability/conductivity^23^ and unpropped fracture compressibility^24^ evaluations are provided elsewhere.
7. The fractured core plug sample was saturated with formation (dead) oil through application of a constant oil pressure of 1500 psi for 5 days—the fractured core plug sample was evacuated for 48 h prior to oil saturation to remove the air in the matrix and fracture.
8. The oil was thoroughly removed from the dead volumes and the fracture after oil saturation and before the gas injection tests to minimize the error on the original oil-in-place (OOIP) calculations.
9. The core plug sample was removed from the coreholder and the mass was measured to allow the calculation of oil saturation and OOIP using helium porosity data obtained previously (step 3).
10. The unpropped fracture permeability (and compressibility) was measured with liquid (formation oil) under loading and unloading paths using the steady-state liquid flow technique. The liquid permeability values were measured after each incremental increase in axial/radial stress conditions and repeated within 12-h time intervals under similar experimental conditions until a constant value (within experimental error range) was reached. To examine the impact of flow rate on fractured oil permeability, oil was injected into the fractured core plug sample at different flow rates (0.5–1.5 cc/min) under varying effective stress conditions (500–3800 psi). Darcy’s law was used to determine the liquid (oil) permeability of the fractured core plug under each flow rate and effective stress conditions.
11. CO_2_ or simplified lean gas were injected into the core plug sample at a constant pressure, followed by a soaking period (1 h) for each cycle of the HNP process—during CO_2_ HNP cycles, liquefied CO_2_ was injected into coreholder from the inlet end with a constant pressure of 1340 psi for 1 h until reaching the miscible condition, while during the lean gas HNP cycles, simplified lean gas was injected into the coreholder from the inlet end with a constant pressure of 1280 psi for 1 h. Prior to CO_2_ HNP experiment, the minimum miscibility pressure (MMP) between the Duvernay (dead) oil and CO_2_ was estimated from the slim tube simulation and found to be about 1028 psi. This MMP was also consistent with the values predicted from correlations. Using the slim tube simulation results, a constant CO_2_ injection pressure of higher than 1028 psi (i.e. 1340 psi) was employed in the CO_2_ HNP experiments to avoid immiscible conditions. Similar experimental conditions were applied during the lean gas HNP process for better comparison of the recovery performance.
12. The gas (e.g. CO_2_ or lean gas)/oil mixture was produced by decreasing downstream pressure to atmospheric pressure for a specific period of time (0–4 h, depending on the cycle) until the point where recovery factor was lower than 1–2% of OOIP.
13. Produced gas and oil were sub-sampled after selected steps for compositional analysis.
14. Steps 11–13 were repeated for multiple cycles, measuring oil and gas production for each step, and oil/gas composition after selected steps—in total, six and four cycles of CO_2_ and lean gas HNP tests were performed herein, respectively.

During the CO_2_ HNP process, initially, four cycles of HNP experiments were conducted under the experimental conditions described herein. However, in order to evaluate the impact of varying experimental conditions on the oil recovery, a cycle (#5) with shorter injection time (0.5 h), no soaking and a shorter production time (1 h) was employed. Thereafter, keeping the injection and production conditions identical to those of cycle #5, an additional cycle (#6) was conducted with 1 h of soaking to further evaluate the effect of soaking time on recovery performance. During the simplified lean gas HNP process (cycle #1–4), similar experimental conditions were applied as the first four cycles of CO_2_ HNP process described above (e.g. 1 h of gas injection, 1 h of soaking and 4 h of production) for direct comparison of the EOR performance using the two different gases.

## Results

### CO_2_ vs. simplified lean gas HNP

**Figure 3:**
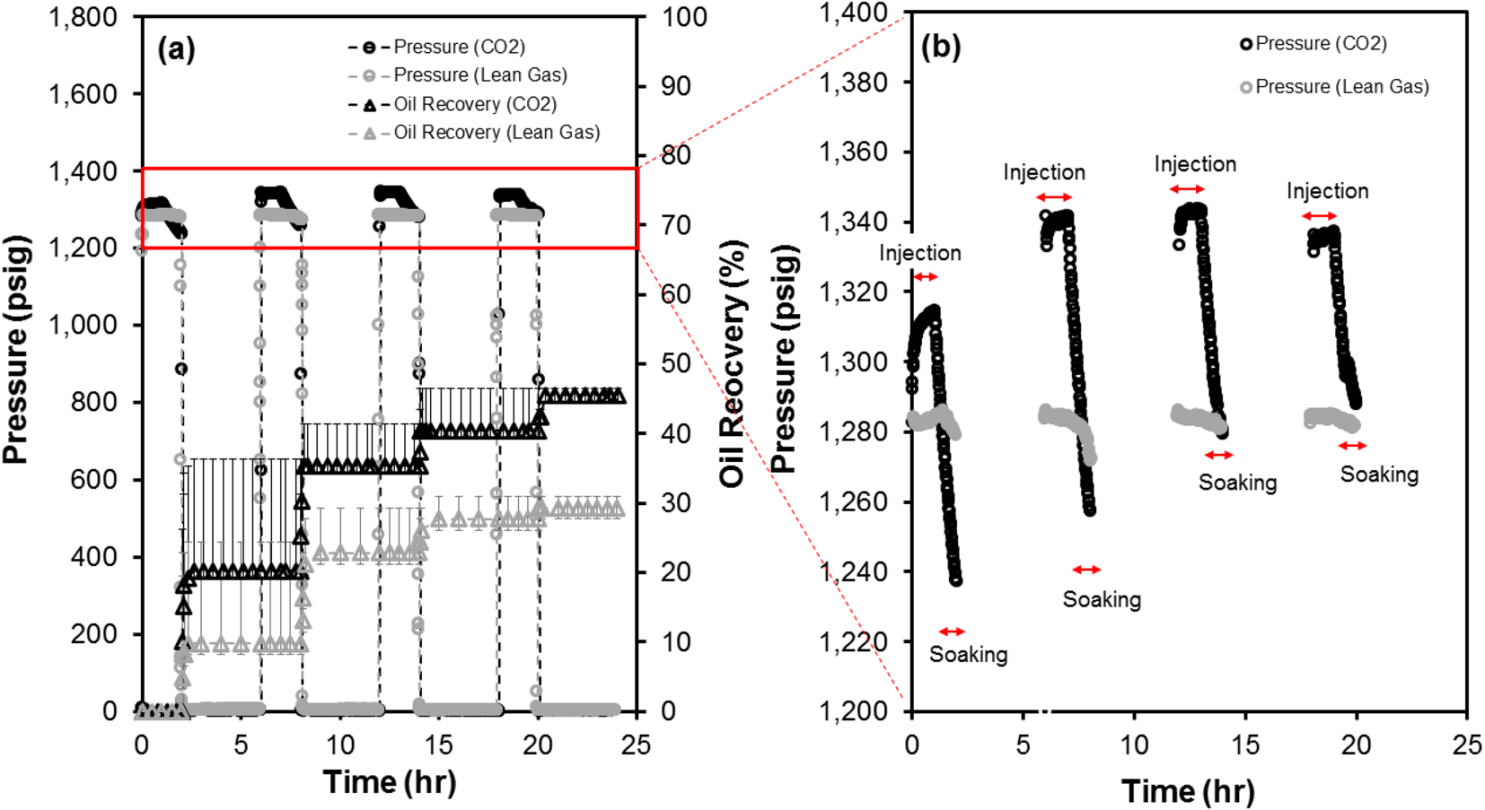
** (a,b)** Oil recovery and injection/production pressures (4 cycles) as a function of (elapsed) time for the CO_2_ and lean gas HNP cycles performed on twin core plug samples **(a)**. CO_2_ data are adopted from Song et al.^28^ while lean gas data are generated in this work. Pressure decay profiles for CO_2_ and lean gas in an enlarged view **(b)**.

**Figure 4:**
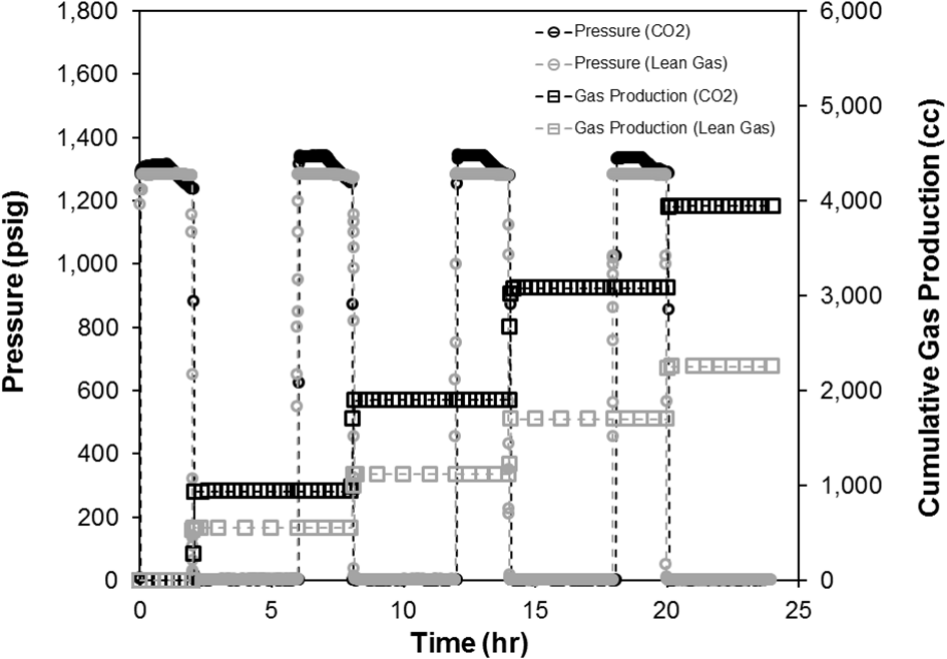
Cumulative gas production and injection/production pressures as a function of (elapsed) time for the CO_2_ and lean gas HNP cycles performed on the twin core plug samples (4 cycles). CO_2_ data are adopted from Song et al.^28^ while lean gas data are generated in this work.

Based on the measured oil recovery after each cycle (Fig. 3a), it is evident that the majority of oil recovery (up to 80%) occurs during the first two cycles either for the CO_2_ or lean gas HNP (4 cycles in total). Further, time-dependent pressure data (Figs. 3b, 4) indicate that there is a pressure drop during each soaking period for CO_2_ HNP tests, likely due to CO_2_ dissolution into the formation oil. However, a very minor pressure drop is evident for the lean gas HNP experiment. For cycles #1 to #4 of the CO_2_ HNP, which were conducted under identical experimental conditions, the rate and magnitude of the observed pressure drop decrease with each cycle. The impact of operational conditions on the performance of core-based HNP (with CO_2_, as example) is discussed in Supplementary Appendix S3 online.

The observed pressure drops are partly controlled by the effective diffusion/dispersion process (next section), which is a form of dissolution, as proposed before for other gas/liquid diffusion/dispersion models (e.g. Crank 1975^33^). The substantial pressure drops for CO_2_ are due to larger CO_2_ effective diffusion/dispersion coefficient according to the simulation results, that are generally in agreement with the orders of CO_2_ and lean gas (i.e. CH_4_) dissolution in typical dead oil samples (2–3 times^34^ higher for CO_2_ compared to CH_4_).

There are still considerable experimental challenges associated with the accurate determination of oil recovery during cyclic gas injection in low-porosity rocks. The experimental error and uncertainty in recovered oil volumes and recovery factors are discussed in detail in Supplementary Appendix S4.

### History matching: post experiments

Several parameters were modified in the base simulation models to match the measured oil recovery profiles. History matching was conducted manually to achieve the ‘best-match’ because there were numerical challenges to complete the history matching using other assisted approaches (e.g. Adaptive Differential Evolution^35^ and Bayesian Optimization methods^36^). The latter observation is attributed to (1) the small grid block thickness (as small as 0.003 cm), (2) the complexity of the compositional simulation for near-miscible conditions, (3) large pore volumes and transmissibility differences between the simulation cells (due to the presence of fracture), and (4) most importantly, including effective dispersion/diffusion coefficient as an input parameter for simulations.

**Figure 5:**
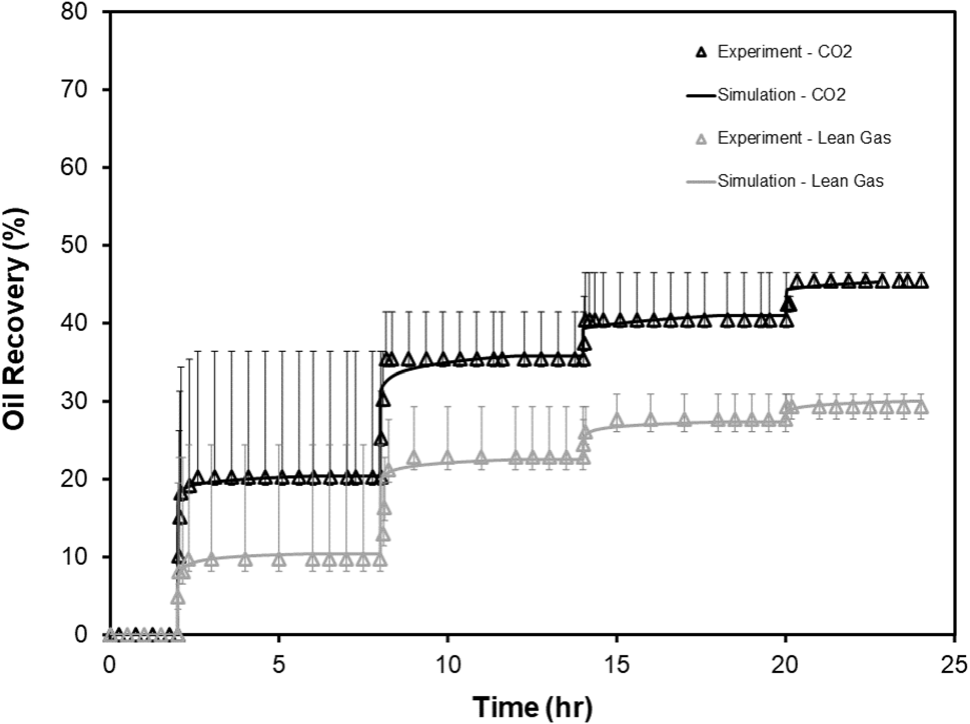
Comparison between experimental and simulated oil recovery as a function of time for the CO_2_ and lean gas HNP cycles (#1 to #4). Note the last two cycles (#5 and #6; see Supplementary Appendix S3 online) for CO_2_ HNP are not simulated because (1) they were not simulated before the experiments and (2) they did not result in significant additional recovery. CO_2_ data are adopted from Song et al.^28^ while lean gas data are generated in this work.

The preliminary (manual) history matching results indicate that a reasonable match can be achieved for both CO_2_ and lean gas HNP processes (Fig. 5) by changing the effective diffusion/dispersion coefficients and increasing the fracture width for both models (Supplementary Table S3 online). For the CO_2_ injection, it was assumed that D_oil_ = 0.1 D_gas_.

For the CO_2_ HNP test (Fig. 5), the sharp increase in oil recovery observed during the first cycle can only be matched with the simulator by allowing more CO_2_ to flow into the system. The sharp increase could be caused by additional mixing of CO_2_ and the (dead) oil at the injection face of the core. An attempt was made to test this hypothesis with the numerical model by adding an additional set of vertical grid blocks on the injection side of the core with large permeability and adjustable pore volumes. However, this approach resulted in instabilities in the compositional simulations. A simpler approach was then tried, which involved increasing the fracture width to permit enough CO_2_ to flow into the core plug sample.

For the lean gas HNP simulations (Fig. 5), the PVT model was re-constructed from 7 pseudo-components (applied in CO_2_ HNP simulation) to 11 pseudo-components where C_1_ and C_2_ were considered to be separate components in the simulation. The initial matrix permeability was slightly adjusted to about 30 nd due to the high effective stress (1300 psi; 8.96 MPa) applied prior to the start of gas injection. Similar to the CO_2_ HNP test analysis, the fracture width of the core plug was also increased to allow more gas to flow into the system. For the simulation results, it was found that the slope of the oil recovery curve was sensitive to the effective diffusion/dispersion coefficients of other components. Therefore, the coefficients of a few other components were also modified for both the CO_2_ and lean gas injection cases in order to achieve the “best-match” between experiments and simulation model.

The history-matched model indicates that, while the oil recovery for cycle #1 can be matched reasonably well, there is a slight mismatch between measured and simulated oil recoveries at the beginning of cycles #2 to #4 for both CO_2_ and lean gas. The observed mismatch could be attributed to inherent differences between the nature of experiment and the simulation. In numerical simulation, the produced oil contribution to improved recovery is accounted for as soon as the oil is produced from the core in the simulation. During actual experiments, however, the produced oil may become ‘trapped’ in the facture or dead volume of the experimental setup instead of flowing out into the sample collector. The remaining oil content, that is associated with an earlier (i.e. previous) cycle, may therefore become part of the produced oil in accompanying subsequent cycle, leading to a sharp and sudden (as a result of abrupt pressure drop) increase in (experimental) recovery profiles at the beginning of cycles #2 to #4 for CO_2_ and lean gas HNP processes.

**Figure 6:**
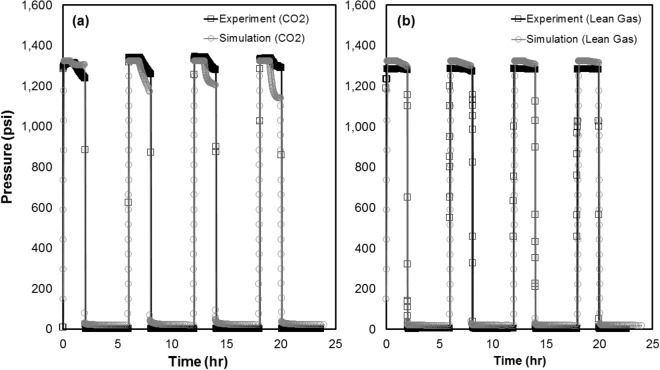
Comparison between experimental and simulated pressure decay profiles for CO_2_ **(a)** and lean gas **(b)**.

Note using a ‘manual’ history matching process (as opposed to an ‘assisted’), it was not possible to achieve the “best-match” for all pressure decay profiles for such small system including diffusion/dispersion process. As such, the “best-match” model remains only an approximation. However, while an approximation, the simulated pressure decay profiles (Fig. 6a,b) are capable of predicting higher pressure decay for CO_2_ than lean gas during the soaking period (in agreement with the experimental data), highlighting the importance of the effective diffusion/dispersion process on cyclic gas injection. Due to the possibility of gas compression in the system as a result of the nature of the experiments (i.e. higher gas volumes injected/produced over larger drawdowns compared to intact cores), the recorded gas volumes were treated with caution, and therefore, the cumulative gas recovery was not considered in the history matching process.

## Discussion

The previous laboratory studies represent a step change in the thinking around experimental evaluation of core-based HNP process through suggesting alternative flow geometries^7,20^, use of large-scale samples/fractures^7^ and combining ‘outside-of-coreholder’ drilling and advanced core-based hydraulic fracturing under stress inside the coreholder to induce fractures^8^. However, the current work is unique due to (1) the proposed methodology used for creating fractures under stress and (2) its integrated experimental and simulation approach in which a compositional model is calibrated to laboratory data (porosity, matrix/relative permeability, etc.). To our knowledge, this study is the first to fully couple fracturing under stress and EOR approaches with predictive fine-tuned simulation models for the purpose of comparing the cyclic gas injection performance of CO_2_ and lean gas—two primary components of greenhouse gases—in fractured hydrocarbon systems. Compared to previous experimental approaches, the method proposed herein reproduces ‘near-fracture’ conditions better because (1) fractures are created under stress along the weakest mechanical plane, (2) the actual sequence associated with a HNP cycle is mimicked (injection, soaking and production), (3) gas and liquid flow occur through an actual induced fracture (analogous to a hydraulic fracture created ‘in-situ’ during a stimulation treatment), and (4) “in-situ” stress, and stress variation regimes during injection, soaking and flow are reproduced. Recovery factors of 45% and 30% of OOIP were obtained after four cycles of HNP experiments performed on twin Duvernay Shale core plug samples using CO_2_ and lean gas, respectively. While there may be discrepancies between laboratory- and field-scale recovery factors due to differences between laboratory and field conditions (e.g. dead vs. live oil^8^, reservoir heterogeneity, etc.), the observed difference between CO_2_ and lean gas recovery is in agreement with those reported previously in the literature using “flow-through-matrix” or “flow-around-matrix” scenarios (Table 1). Very importantly, however, in contrast to previous laboratory studies performed on intact/fractured cores (Supplementary Table S1 online), these ultimate recovery factors could be achieved in only 24 h as a result of 1) employing a fractured core plug (i.e. higher surface area) and 2) optimal simulation-aided experimental design. Our integrated workflow therefore provides the opportunity for repeating multiple cycles over a relatively short period of time on a given core plug sample in the laboratory. This in turn provides significant time/cost-savings compared to previous approaches, allowing important results to be turned around much more quickly. Note, using the simulations performed prior to the tests, the experimental time scales were selected with the criteria of achieving higher oil production/recovery while shortening the total duration of the injection-soaking-production cycles (not achieving e.g. complete pressure equilibrium after each cycle, etc.).

**Table 1:** Comparison of core-based HNP experiments with CO_2_ and lean gas in tight oil/gas formations.

Formation	Porosity (%)	Permeability (md)	Method	Gas type	Oil recovery (%)	References
Middle Bakken (USA)	4.4–5.4	0.008–0.1	”Flow-around”	Lean gas^a^	> 90	^37^
				CO_2_	> 90	
Lower Bakken (USA)	3.8	0.005	”Flow-around”	Lean gas^a^	27	
				CO_2_	32	
Wolfcamp (USA)	8.5	0.0003–0.0005	”Flow-around”	CO_2_	65	^14^
				Lean gas^b^	30–40	
Middle Bakken (USA)	4.5–8.1	0.002–0.04	”Flow-around”	CO_2_	~ 95	^38^
				Lean gas^a^	~ 95	
				Lean gas^b^	92	

^a^Simplified lean gas (85% CH_4_ + 15% C_2_H_6_) is used in the tests.

^b^Pure lean gas (100% CH_4_) is used in the tests.

Besides improved recovery, one of the advantages of using greenhouse gases (e.g. CO_2_ and lean gas) as solvents is that a portion of the injected gas could be sequestered/trapped in the reservoir via dissolution in the oil, adsorption on the rock surface, amongst other mechanisms. Optimizing the balance between improved hydrocarbon liquid recovery and underground CO_2_ storage is among future challenges ahead of unconventional hydrocarbon industry due to suppressed commodity prices and associated environmental concerns. Thanks to its fast throughput, one of the advantages of the ‘flow-through-frac’ method, proposed herein, is that experimental designs and conditions leading to this optimal balance, which in turn can be used to inform field testing, can be determined quickly. For the CO_2_ HNP experiments performed herein, only a minor fraction (about 25 cc) of CO_2_ was dissolved in the Duvernay (dead) oil under the prevailing conditions compared to the large amount of injected CO_2_ (> 5000 cc). The small dissolution is primarily due to low production pressure (i.e. atmospheric) used in the current experiments, leading to low CO_2_ solubility. Designs to achieve the optimal balance of improved hydrocarbon recovery and underground greenhouse gas storage will be discussed in future studies.

## Samples

### Rock samples

The selected core plugs for this study were twin samples obtained from the same well and depth. These samples (1.5″ diameter, 2″ length) were drilled parallel to bedding from a 2/3 slabbed core obtained from a vertical well drilled into low-permeability intervals of the Duvernay Formation (Alberta, western Canada). A comprehensive series of geochemical, petrophysical and geomechanical analyses were previously performed on sample pieces and twin core plug samples obtained from the same well and depth^29,39^, serving as an important reference point for the current study. The analyzed samples have the following geochemical and petrophysical properties based on the sub-samples taken from the vicinity of these two core plugs: total organic carbon (TOC) content: 4.26 wt.%; clay content: 33.3%; quartz content: 44.7%; carbonate content: 12.4%; helium porosity: 2.1–3.3%; slip-corrected gas (N_2_) permeability (1.25·10^–4^ md; 900 psi effective stress). Detailed descriptions of the experimental procedures for the determination of TOC content, mineralogical composition, helium porosity, and slip-corrected gas (N_2_) permeability are provided elsewhere^22^.

The core plug samples used in this study were analyzed under “as-received” conditions. “As-received” in the context of this work means that the sample was tested without any further treatment (e.g. cleaning/drying) in the laboratory after drilling.

### Rock sample preparation: fracturing under stress

The representativeness and repeatability of the fracture creation procedure is one of the main challenges associated with experimental HNP studies. The ‘quality’ of the induced fractures is important for core-based HNP data evaluation and history-matching. The assumption of the cubic geometry, that is the basis of the data evaluation and simulation for fractured cores, can be violated depending on the roughness and orientation of the induced fractures. Larger errors in property estimates are associated with deviated fractures (from the core axis) with rougher surfaces.

In this work, the fractures were created along cemented laminations (bedding planes) of core plugs. Cemented laminations are commonly formed parallel to the bedding planes that are perpendicular to the direction of sedimentation, creating planes of weakness. Unlike creating “smooth” saw-cut surfaces, the creation of fractures along weak planes under stress ensures that the fracture conditions are representative of the subsurface fracturing process and can be considered relatively repeatable.

**Figure 7:**
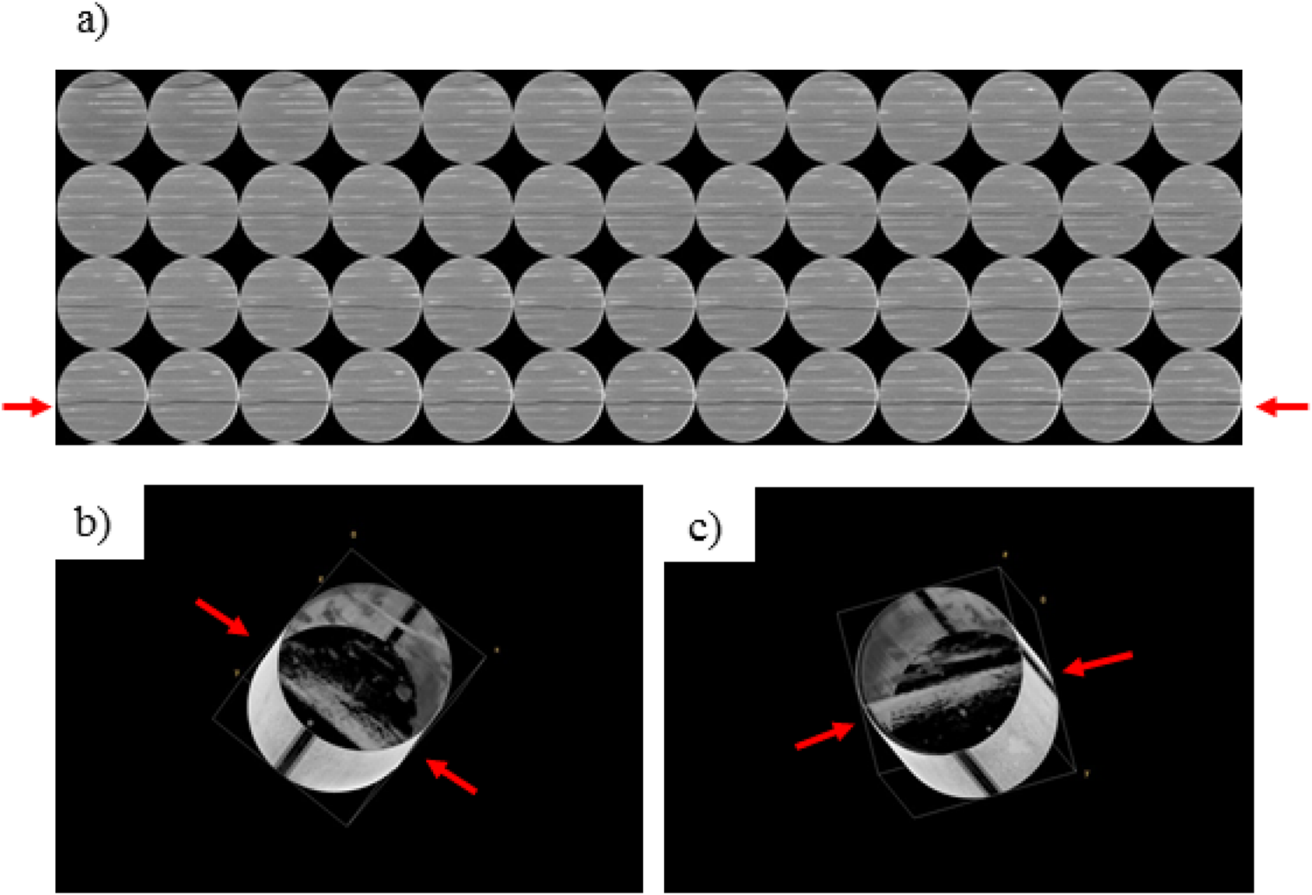
CT scanning images of 2D and processed 3D views of Duv 1–1: **(a)** 2D, top to bottom; **(b)** 3D, top to bottom; **(c)** 3D, bottom to top. A primary cemented lamination that is partly fractured is highlighted with the red arrows **(a–c)**. There is a secondary fracture close to the edge **(a,b)** that is not continuous along the core axis. Drafted using ImageJ (version 1.53a, https://imagej.net/).

To increase the representativeness and repeatability of the ‘flow-through-frac’ technique, preferentially, those core plugs that contain a visible laminations and/or cemented micro-fracture should be drilled from the full core (or 2/3 slabbed core) for the tests (Fig. 7). As an example, for Duv 1–1, a series of CT scans were collected in a commercial laboratory to investigate the presence of laminations and/or pre-existing micro-fractures inside the core plug. A primary micro-fracture was detected along the length of the core plug sample (Figs. 7a–c). This micro-fracture, which extends from top to bottom of the core plug, occurs along a mechanically weak plane which is prone to fracturing under differential stress.

### Fluids

The formation (dead) oil sample was collected from an active producing field in the Duvernay Formation (western Canada). The formation oil was fully dewaxed with 0.1 micron filters at 25 °C in a commercial laboratory prior to experiments. The analyzed oil sample has the following physical properties (25 °C, 14.7 psi): density: 0.823; viscosity: 2.043 cP; and compressibility: 6.5·10^–6^ psi^−1^. The CO_2_ gas used in the CO_2_ HNP experiments was at research grade with a purity of 99.998%, and the simplified compositional lean gas (80% CH_4_ + 20% C_2_H_6_) used in the lean gas HNP experiment was prepared by a commercial vendor in Calgary, Alberta, Canada.

The objective in this research was to evaluate the proof-of-concept experiments for cyclic gas injection at ‘near-fracture’ conditions in *locally* *depleted* tight oil reservoirs (i.e. with large drawdowns in near-fracture region) that presumably completed the primary recovery process. As such, dead oil samples (as opposed to live oil) were used for HNP experiments without performing any primary recovery tests prior to the HNP tests.

## Supplementary Information

Supplementary Information.
