# Supplementary material for: Experimental and computational evaluation of cyclic solvent injection in fractured tight hydrocarbon reservoirs
Source: Sci Rep. 2021 May 4;11:9497. doi: 10.1038/s41598-021-88247-y (PMC8097080; doi:10.1038/s41598-021-88247-y)
Supplement: Supplementary file 1 — Supplementary Information. [file 41598_2021_88247_MOESM1_ESM.docx]

**Supplementary Material**

Experimental and Computational Evaluation of Cyclic Solvent Injection in Fractured Tight Hydrocarbon Reservoirs

Amin Ghanizadeh*, Chengyao Song, Hamidreza Hamdi, and Christopher R. Clarkson

Department of Geoscience, University of Calgary

*Corresponding author: aghaniza@ucalgary.ca

**Supplementary Table S1 (online**). Literature review of core-based HNP experiments in tight oil/gas formations. Very few laboratory-based studies have been conducted on tight hydrocarbon systems with permeability values down to the nanodarcy range^7-8,14-17,19, 43^. The laboratory methodologies are primarily focused on intact (‘unfractured’) core plug samples using either the ‘flow-through-matrix’^10,12,17-19,40-42^ or ‘flow-around-matrix’^14-16,21,37-38,43-44^ schemes, which do not realistically represent the fracture-matrix contact or sequences associated with a typical cyclic solvent injection (HNP) process. Modified after Song et al.^28^

| Formation | Sample Condition | Gas Type | Porosity (%) | Permeability (md) | Experimental Method | Cycle No. | Test Time (h) | Final Recovery (%) | Ref. |
| --- | --- | --- | --- | --- | --- | --- | --- | --- | --- |
| Middle Bakken  (USA) | Intact | CO_2_ | 4.4-5.4 | 0.008-0.1 | “Flow-around” | 3 | 24 | >90 | [37] |
| Middle Bakken (USA) |  | Lean gas^(1)^ |  |  | “Flow-around” | 3 | 24 | >90 |  |
| Lower Bakken  (USA) | Intact | CO_2_ | ~3.8 | ~0.005 | “Flow-around” | 3 | 24 | 32 |  |
| Lower Bakken (USA) |  | Lean gas^(1)^ |  |  | “Flow-around” | 3 | 24 | 27 |  |
| Bakken  (USA) | Intact | CO_2_ | 18.6-23.1 | 0.3-0.8 | “Flow-through” | 4-6 | 40-60 | 43-63 | [18] |
| Wolfcamp  (USA) | Intact | CO_2_ | 7.5 | 0.0002 | “Flow-around” | 7 | 84 | ~35-70 | [15] |
| Wolfcamp (USA) | Intact | CO_2_ | ~8.5 | 0.0003-0.0005 | “Flow-around” | 6 | 78 | ~65 | [14] |
|  |  | N_2_ |  |  | “Flow-around” | 6 | ~145 | 35-50 |  |
|  |  | CH_4_ |  |  | “Flow-around” | 6 | ~145 | ~30-40 |  |
| Bakken  (USA) | Intact | CO_2_ | 4.5-8.1 | 0.002-0.04 | “Flow-around” | 5 | 70-96 | 60-100 | [21] |
| Unknown | Intact | CO_2_ | 14.6 | 0.3 | “Flow-through” | 1-4 | 6.5-8 | 21-41 | [12] |
| Montney  (Canada) | Intact | CO_2_ | 14.0-15.5 | 2-5 | “Flow-through” | 8 | 72 | 66-77 | [40] |
| Eagle Ford  (USA) | Intact | CO_2_ | 7.5 | 0.0003 | “Flow-through” | 6 | 78 | 61 | [19] |
| Baikouquan  (China) | Intact | CO_2_ | 9.3 | 0.9 | “Flow-through” | 3 | N/A | 37 | [10] |
| Junggar  (China) | Intact | CO_2_ | 11.8-13.2 | 0.03-0.5 | “Flow-through” | 8 | >96 | 16-28 | [41] |
| Eagle Ford (USA) | Intact | N_2_ | 5.0-7.7 | ~0.0005 | “Flow-through” | 8 | 384-704 | 40-45 | [17] |
| Eagle Ford (USA) | Intact | N_2_ | 9.7-10.6 | 0.0003-0.0004 | “Flow-around” | 12 | 12-288 | 18-25 | [16] |
| Yanchang (China) | Intact | N_2_ | 10.2-10.7 | 0.2-0.4 | “Flow-through” | 5 | ~50-90 | 14-17 | [42] |
| Wolfcamp (USA) | Intact | CH_4_ | 6-8 | 0.003-0.0005 | “Flow-around” | 8 | ~96 | 49 | [43] |
| Middle Bakken (USA) | Intact | Lean gas^(1)^ | 4.5-8.1 | 0.002-0.04 | “Flow-around” | N/A | 24 | 95 | [38] |
| Eagle Ford (USA) | Intact | CH_4_ | N/A | N/A | “Flow-around” | 8 | 244 | 43-50 | [44] |
| Haynesville Shale (USA) | Fractured | Lean gas ^(2)^ | 8 | 0.0006-0.0009 | “Flow-through” | 7 | 230-350 | 35-50 | [8] |
| Montney/Duvernay (Canada) | Fractured | Lean gas ^(3)^ | 6 | ~0.0008 | “Flow-through” | 7 | ~105 | ~35 | [7] |

^(1)^ Composition = 85% CH_4_ + 15% C_2_H_6_

^(2)^ Composition = 91% CH_4_ + 4.4% C_2_H_6_ + 0.4% C_3_H_8_ + 2.3% CO_2_ + 1.1% N_2_

^(3)^ Composition = 77.6% CH_4_ + 21.6% C_2_H_6_ + 0.8% other components

**Supplementary Table S2 (online).** Composition of the simulated (Duvernay) dead oil samples used in the simulations. For the experiments, the same dead oil sample was used for both CO_2_ and lean gas HNP tests. However, for simulation, the PVT model was slightly modified for better history matching of CO_2_ and lean gas results. CO_2_ data are reproduced from Song et al.^28^ while lean gas data are generated in this work.

| **Components** | **Molar Percentage (%)** |
| --- | --- |
| **CO_2_ HNP** | |
| CO_2_ | 0.008 |
| N_2_-C1 | 0.277 |
| C2-C4 | 2.060 |
| C5-C7 | 28.890 |
| C8-C12 | 39.221 |
| C13-C19 | 16.812 |
| C20+ | 12.732 |
| **Lean Gas HNP** | |
| CO2 | 0.008 |
| N2-C1 | 0.334 |
| C2 | 0.527 |
| C3 | 1.251 |
| iC4-nC4 | 2.286 |
| iC5-cC5 | 3.773 |
| C6 | 6.61 |
| C7-C9 | 37.882 |
| C10-C14 | 26.058 |
| C15-C23 | 14.199 |
| C24+ | 7.072 |

**Supplementary Table S3 (online).** The parameters used in the history matching process to achieve the “best-match” between experiments and simulations.

|  | **CO_2_ HNP** | **Lean Gas HNP** | |
| --- | --- | --- | --- |
| Parameter Parameter | Value | Parameter Parameter | Value |
| *D_g-CO2_* | 0.005 cm^2^/s | *D_g-CO2_* | 0.00042543 cm^2^/s |
| *D_g-N2-C1_* | 0.0027 cm^2^/s | *D_g-N2-C1_* | 0.00092472 |
| *D_g-C2-C4_* | 0.0014 cm^2^/s | *D_gC2_* | 0.00045423 |
| *D_o-CO2_* | 0.0005 cm^2^/s | *D_g-C3_* | 0.00021959 |
| *D_o-N2-C1_* | 0.00027 cm^2^/s | *D_g-C4_* | 0.00004503 |
| *D_o-C2-C4_* | 0.00014 cm^2^/s | *D_o-CO2_* | 0.000011 |
| Fracture width | 0.015 cm | *D_o-N2-C1_* | 0.000020 |
|  |  | *D_oC2_* | 0.000006 |
|  |  | *D_o-C3_* | 0.000003 |
|  |  | *D_o-C4_* | 0.000001 |
|  |  | Fracture width | 0.0067 cm |

**Supplementary Fig. S1 (online).** Experimental workflow for core-based HNP experiments in fractured core plug samples

**
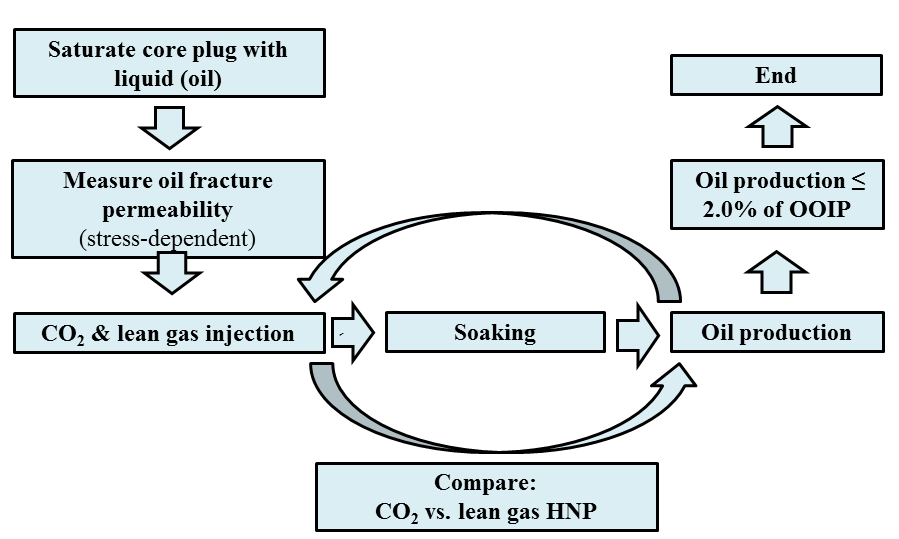
**

**Supplementary Fig. S2 (online).** Phase envelope of the simulated (Duvernay) dead oil sample. Note the P-T diagram belongs specifically to (Duvernay) dead oil sample for CO2 test with a smaller number of components. Similar P-T diagram is expected for lean gas scenario because both dead oil samples are based on an identical equation of state (EOS) that has been tuned differently. Reproduced from Song et al^28^ and drafted using tNavigator (version 20.1, <https://rfdyn.com/tnavigator/>).


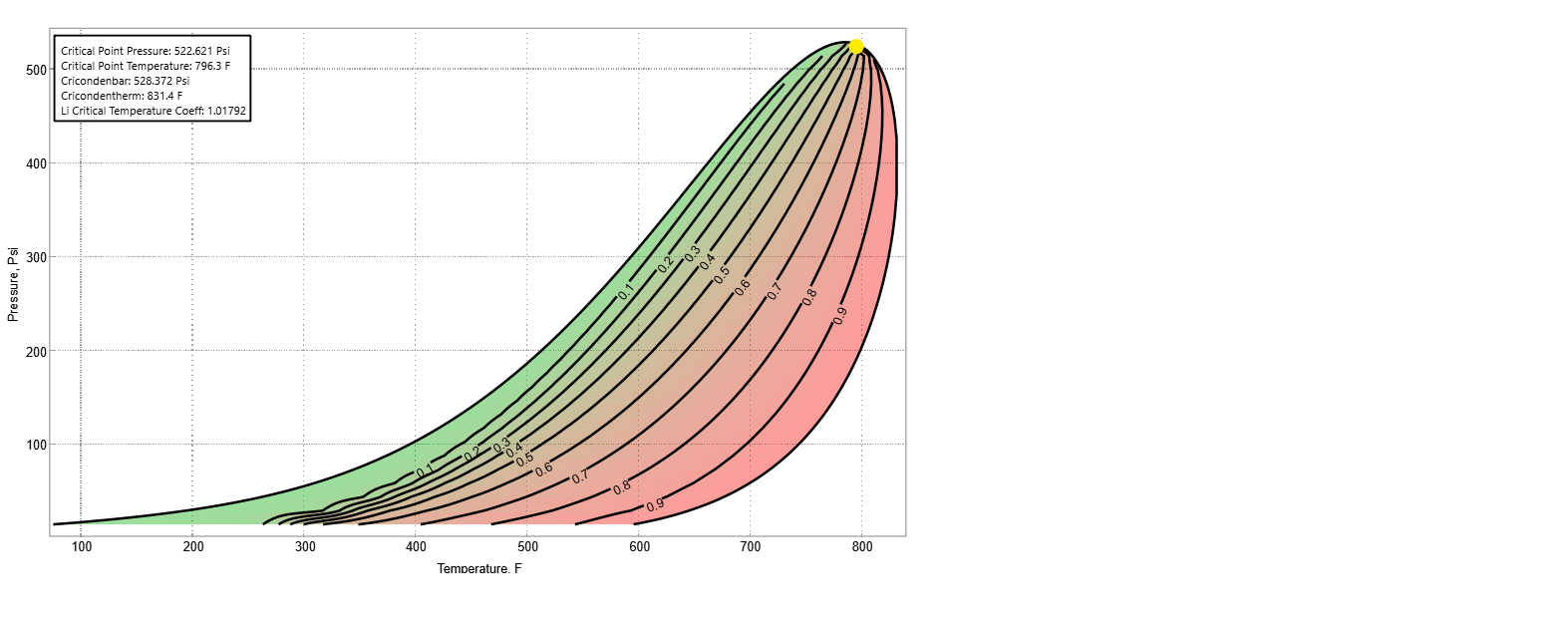


Figure S1 — Phase envelope of the simulated (Duvernay) dead oil sample.

**Supplementary Fig. S3 (online**). Snapshot of 3D pressure variation after injecting CO_2_ and lean gas into the fractured core plug. Reproduced from Song et al^28^ and drafted using tNavigator (version 20.1, <https://rfdyn.com/tnavigator/>).


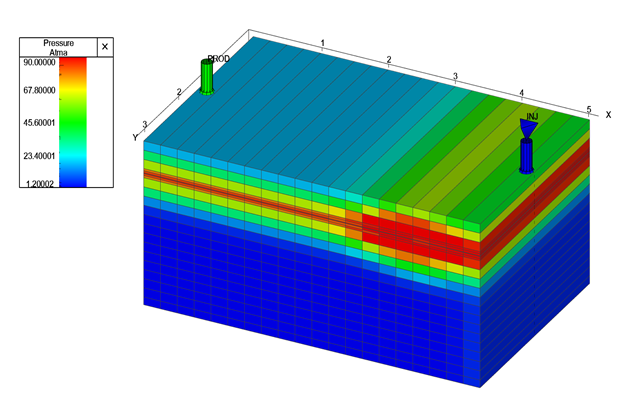


**Supplementary Appendix S1 (online).** Theory of HNP Process

When injecting solvent (e.g. CO_2_) into tight oil and liquid-rich gas formations, a series of physical and chemical processes are expected to occur in the core plug sample depending on whether *miscible* or *immiscible* injection conditions are observed. Under miscible conditions, the primary mechanisms include 1) swelling of the oil in the matrix^7,12,45,4647^ and 2) interfacial tension (IFT) reduction between gas and liquid^7,46,48^, which in turn facilitate oil flow within the fractures, leading to improved oil recovery. However, under immiscible injection conditions, the primary mechanisms are 1) interfacial tension (IFT) reduction, 2) oil viscosity reduction (the dissolution of CO_2_ into the crude oil), 3) lighter components extraction^7,12,46^, and 4) solution gas drive. For 3), the injected solvent partly dissolves into the oil under immiscible/miscible conditions, extracting the light to intermediate components to enrich the injected gas phase when re-produced from the reservoirs subsequently^7,49^. For 4), the solution gas is released dynamically during production period due to the difference between *surface* vs. *subsurface* pressure and temperature conditions, forcing the oil out of matrix into the fractures^18^. During the lean gas HNP, a few additional mechanisms including reservoir pressure maintenance, residual oil shrinkage by lighter components extraction, lean gas (e.g. methane) adsorption and gas trapping effect may also occur^4,7,50^. Details of fundamental theories that describe HNP processes in tight hydrocarbon systems are explained elsewhere^6,51–56^.

**Supplementary Appendix S2 (online).** Stress-dependent Fracture Liquid (Oil) Permeability

During the loading cycle (**Fig. AS2**), fracture liquid (oil) permeability decreases continuously, up to about 1.5-2 orders of magnitude (from about 2 to 0.09 md for Duv 1-1, from about 150 to 11 md for Duv 1-2) with increasing effective stress (500-3800 psi). In addition, under any given effective stress condition, fracture liquid (oil) permeability increases slightly with increasing liquid flow rate (0.5-1.5 cc/min). During the unloading cycle (**Fig. AS2**), fracture liquid (oil) permeability increases continuously, up to about 5-6 times (from 0.09 to 0.56 md for Duv 1-1, from 11 to 50 md for Duv 1-2), with decreasing effective stress (3800 to 500 psi). Fracture liquid (oil) permeability values measured during the unloading cycle are consistently lower than those measured during the loading cycle, resulting in hysteresis between the loading-unloading cycles (Fig. A3). Similar observations (i.e. hysteresis) were previously observed for fracture permeability data measured with gas (N_2_) for one of the fractured core plug sample (Duv 1-2). For Duv 1-2, the fracture *gas* permeability is consistently (up to about 3 times) higher than fracture *liquid* permeability under identical effective stress conditions regardless of the liquid (oil) flow rate.

**
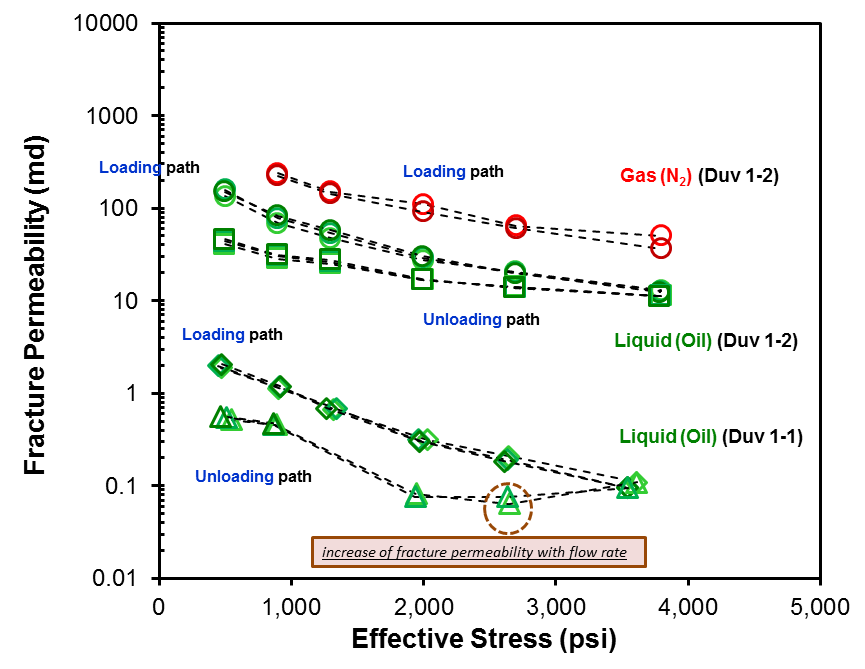
**

**Supplementary Fig. AS2 (online**)**.** Stress-dependent fracture liquid (oil) and gas (N_2_) permeability values measured under loading and unloading cycles on the analyzed Duvernay core plug samples. The fracture gas (N_2_) permeability values are measured with mean pore pressures of 15.2 psig and 16.0 psig, respectively. Under each effective stress condition, the fracture liquid (oil) permeability is measured with increasing flow rate (0.5-1.5 cc/min). The fracture liquid (oil) permeability increases slightly with increasing liquid flow rate (0.5-1.5 cc/min).

**Supplementary Appendix S3 (online).** Impact of Operational Conditions on Core-based HNP Performance

In-situ flow of mobilized oil from the matrix to the fractures in tight oil formations can be sensitive to production conditions (e.g. time, pressure, etc.). According to previous studies^47^, production time can be optimized to increase enhanced recovery during the HNP process in fractured tight hydrocarbon reservoirs. To examine the influence of operational parameters on oil recovery, the last two cycles of CO_2_ HNP (cycles #5 and #6) were conducted under different experimental conditions from those of cycles #1 to #4. Comparing cycles #4 and #5, it is evident that the recovery factor achieved after cycle #5 (about 2% of OOIP) is significantly lower than that of cycle #4 (about 5% of OOIP). This observation is partly attributed to the shorter injection time, ‘no soaking’, and shorter production time condition employed in cycle #5. However, it should also be noted that the efficiency of successive cycles in any core-based HNP process is expected to reduce over time. This is mainly because most of the “*in-situ*” oil that can be mobilized and drained toward the fracture face is generally depleted during the early cycles. Further, injecting additional gas into the system can result in larger gas relative permeabilities in the depleted area which can substantially reduce the mobility of the oil phase.

**Supplementary Appendix S4 (online).** Experimental error and uncertainty in recovered oil volumes and recovery factors.

Performing cyclic gas injection tests on low-porosity rocks is technically challenging in laboratory. Due to the small pore volume, the volume of the recovered oil could potentially become as small as the experimental error, posing uncertainty on the estimated recovery factors. The challenge is exacerbated particularly for testing smaller core plugs (e.g., 1.5” diameter, 2” length; such as those used in this study) that are routinely preferred over full cores to avoid impractically long experimental duration.

The uncertainty in the recovered oil volume in each cycle depends not only on the measuring tube/syringe accuracy, but also the ‘trapped’ oil in the downstream dead volume. This is because the oil droplets that are produced from the core plug during each cycle may not necessarily flow out of the (downstream) dead volume immediately, depending on the drawdown pressures and/or capillary effects. If present, these ‘trapped’ oil droplets reside inside the dead volume temporarily, causing the recovered oil volume to be underestimated. However, the ‘trapped’ oil droplets are recovered partly or fully at the beginning of the production stage of the following cycle, depending on the experimental conditions (e.g., drawdown pressures, etc.). The ‘*absolute*’ error associated with the ‘trapped’ oil in the dead volume is expected to reduce after each cycle because 1) less oil is produced in each following cycle, and therefore, less oil is trapped in the dead volume, and 2) the (repeated) gas production stages conducted at the end of each consecutive cycle under high drawdown pressures (~1300 psi herein) are expected to flush the remining oil partly or fully out of the dead volume. As such, the final recovery factors are less affected by the experimental error compared to the cycle-by-cycle recovery factors. In this work, the downstream dead volume was comprised of the volume within a capillary tube (~0.55-0.8 cc) and the dead volume (~0.20 cc) associated with a needle valve (technically inevitable to avoid due to system specifications).

For error evaluation in this work, it is assumed that 1) the downstream dead volume is fully occupied with ‘trapped’ oil at the end of each cycle (max. error calculation) and 2) the ‘trapped’ oil at any given cycle is produced *entirely* in the following cycle due to the large drawdown pressures (~ 1300 psi) applied over a short period to mimic the near-fracture conditions. Therefore, for any given cycle, the (max.) volume of the ‘trapped’ oil is estimated to be equal to the recovered oil volume in the following cycle. Based on the description above, the (max.) error bars for any given cycle have been calculated by adding the volume measurement error (i.e., the measuring tube/syringe accuracy, 0.01 cc) to the volume of the recovered oil in the following cycle (**Figs 3a,b** and **Fig. 5**). Only for the final cycle (i.e., cycle #4), it is assumed that the error is dictated merely by the measuring tube/syringe accuracy (0.01 cc) due to the minimal oil production.

A few laboratory “best-practices” were applied in this work to mitigate the error induced by the dead volume in OOIP and recovery calculations including: 1) the downstream dead volumes were designed to be smaller (~ 0.75-1 cc) than the samples’ pore volumes (~1.2-1.8 cc) for both CO_2_ and lean gas injection tests (using shortened capillaries with shorter inner diameter, placing filling rods inside the tubes is another alternative), 2) the system dead volumes were disassembled and cleaned thoroughly with pressurized gas after oil saturation and before gas injection, and, 3) the production pressures were reduced to highly depleted condition (i.e. atmospheric) to represent the large drawdown at near-fracture region. While precautions 1) and 2) help to reduce the uncertainty in OOIP calculation due to the reduced inaccuracy associated with the dead volumes, precaution 3) ensures the ‘trapped’ oil, if any, is partly or fully produced (i.e. blown out by expanded gas) at the next cycle(s) to mitigate the overall impact on final oil recovery. A few additional precautions will be considered in future works to further mitigate the impact of dead volume on the measured oil recovery. These additional precautions will include: 1) using customized tubes with smaller inner diameters equipped with filler rods to decrease the dead volume and the likelihood of oil trapping and 2) applying vacuum at the end of each cycle for a very short period to evacuate the ‘trapped’ oil in the (downstream) dead volume.
